# Prevalence of obstructive sleep apnoea in acute coronary syndrome patients: systematic review and meta-analysis

**DOI:** 10.1186/s12872-020-01430-3

**Published:** 2020-03-24

**Authors:** Michael R. Le Grande, Alison Beauchamp, Andrea Driscoll, Alun C. Jackson

**Affiliations:** 1 Australian Centre for Heart Health, 75 Chetwynd Street, North Melbourne, VIC 3051 Australia; 2 Faculty of Health, Deakin University, Burwood, VIC 3216 Australia; 3 Melbourne Centre for Behaviour Change, School of Psychological Sciences, The University of Melbourne, Parkville, VIC 3052 Australia; 4 Department of Medicine -Western Health, The University of Melbourne, Parkville, VIC 3052 Australia; 5 Australian Institute for Musculoskeletal Science (AIMSS), St.Albans, VIC 3021 Australia; 6 School of Rural Health, Monash University, Newborough, VIC 3825 Australia; 7 Centre for Quality and Patient Safety Research, School of Nursing and Midwifery, Deakin University, Geelong, VIC 3220 Australia; 8 Centre on Behavioural Health, Hong Kong University, Pakfulam, Hong Kong

**Keywords:** Obstructive sleep apnoea, Prevalence, Acute coronary syndrome, Sleep assessment

## Abstract

**Background:**

Obstructive Sleep Apnoea (OSA) has been recognised as a risk factor for cardiovascular diseases such as hypertension and cardiovascular events such as acute coronary syndrome (ACS). Since it is also known to reduce exercise tolerance, it is important to establish the prevalence of OSA in ACS patients, particularly in those who are commencing cardiac rehabilitation (CR) programs.

**Methods:**

Using PRISMA guidelines a systematic search was conducted in order to identify studies that objectively measured (using polysomnography or portable monitoring) the prevalence of OSA in ACS patients following hospital admission. A data extraction table was used to summarise study characteristics and the quality of studies were independently assessed using the Joanna Briggs Institute Prevalence Critical Appraisal Tool. Meta-analysis of the selected studies was conducted in order to estimate OSA prevalence as a function of the two main methods of measurement, the severity of OSA, and timing of the OSA assessment following ACS hospital admission.

**Results:**

Pooled prevalence estimates of OSA using the “gold standard” polysomnography ranged from 22% for severe OSA to 70% for mild OSA, at any time after hospital admission. Similar prevalence estimates were obtained using portable monitoring, but interpretation of these results are limited by the significant heterogeneity observed among these studies.

**Conclusions:**

Prevalence of OSA following ACS is high and likely to be problematic upon patient entry into CR programs. Routine screening for OSA upon program entry may be necessary to optimise effectiveness of CR for these patients.

## Background

Obstructive sleep apnoea (OSA) describes a syndrome of nocturnal respiratory interruptions resulting in sleep fragmentation, daytime hypersomnolence, and oxyhaemoglobin desaturation, usually unrecognized by the patient [1]. The prevalence of OSA in the general population varies significantly according to variations in diagnostic criteria [2], but is estimated to be close to one in five adults [3]. The risk of OSA rises with increasing body weight; active smoking; diabetes; and age [4] and is associated with elevated risk of coronary heart disease (CHD).

Failure to manage OSA may also be of particular importance in secondary prevention of CHD. There is evidence that cardiac rehabilitation (CR) programs that include an exercise training component may improve cardiorespiratory fitness, lifestyle risk factor management, psychosocial health and potentially reduce the risk of recurrent heart attacks. More specifically, there is convincing evidence for the existence of a dose-response gradient between volume of exercise and clinical outcomes in CR patients [5, 6]. Since it is known to reduce exercise tolerance [7, 8], and therefore volume of exercise, OSA may function as a potential barrier to effective CR. Indeed, a number of studies with acute coronary syndrome (ACS) patients, have demonstrated how continued sleep problems can adversely affect postoperative recovery, morbidity, and quality of life [9–11].

In order to investigate the prevalence of OSA it is important to identify the methods for measuring its prevalence in epidemiological studies. The “gold standard” test for diagnosis of OSA is a conventional, fully supervised, level I polysomnography (PSG) sleep study [12] but since referral for PSG can be difficult, inconvenient, and expensive [13], unattended portable level II to level IV devices are often utilised instead. In addition, the severity of OSA is quantified by the apnoea-hypopnoea index (AHI), which is the number of breathing cessations (apnoea) and reduced airflow (hypopnoea) events per hour. An AHI of 5–15 indicates mild disease, 15–30 indicates moderate disease, and > 30 indicates severe disease [14]. Any estimate of prevalence must clearly indicate which of these objective measures and which AHI cut-offs are used to define OSA.

In addition to the variation in assessment equipment and severity, much of the variability in prevalence estimations is due to variations in timing of the assessment in relation to the ACS event or surgical procedure. In the short-term, physical and emotional recovery is generally faster when percutaneous coronary intervention (PCI) I is performed in comparison to coronary artery bypass graft surgery(CABGS), but this difference usually dissipates by 12-months post-procedure [15]. There is also some evidence that this improvement over time in physical function and more specifically cardiac function, such as improved ejection fraction, is associated with significant reduction of OSA severity over the first 6 months post-procedure [16, 17]. Variations in OSA prevalence would therefore be expected at these key times: pre-procedure (before surgery), post-procedure (in the few days after surgery), recovery (in the immediate 2–8 weeks after surgery, usually when patients are expected to commence CR or long-term (more than 2 months after surgery).

Secondary analysis of CR patient data has indicated that sleep disorders such as OSA have the potential to hamper rehabilitation efforts by affecting treatment adherence, self-efficacy and psychological wellbeing [18]. Until recently [19, 20], OSA has rarely been mentioned as a risk factor to be managed among cardiac patients. The widespread absence of OSA as a target for screening in CR may be due to the relative paucity of prevalence data in this population. Currently, recommendations and decisions for OSA screening in cardiac patients is based on estimates derived from individual disparate studies. Determination of robust estimates of OSA prevalence in ACS patients via the highest level of evidence, a rigorous systematic review and meta-analysis, would therefore be an important and novel contribution to the evidence base.

## Method

We conducted this meta-analysis in accordance with the Preferred Reporting Items for Systematic reviews and Meta-Analyses for Protocols 2015 (PRISMA-P) statement [21]. The checklist of the PRISMA-P guidelines has been included in the supplemental material (Additional file 1).

### Search strategy

We searched the following electronic bibliographic databases: MEDLINE, Embase, CINAHL, the Cochrane Library, and Google Scholar. The search strategy included medical subject heading (MeSH) terms relating to obstructive sleep apnoea and acute coronary syndrome (see Additional file 2). Boolean operators, such as “AND” and “OR”, were used to combine search terms as necessary. The search years covered the period 1995 to April 2019, and there were no language restrictions. This systematic review is registered on Prospero (http://www.crd.york.ac.uk/PROSPERO/display_record.asp?ID=CRD42017057948).

### Inclusion and exclusion criteria

Observational studies which consist of either cross-sectional, cohort or case-control studies of at least *n* = 30 ACS patients (unstable angina, ST elevation myocardial infarction, non-ST segment elevation myocardial infarction) within 6 months of hospital admission were included. Since central sleep apnoea is a common occurrence in addition to OSA in heart failure patients, studies that primarily examined heart failure patients were excluded. Studies that provided a total sample of less than 30 participants were excluded. Also, excluded from the analysis were studies that failed to provide sufficient detail regarding definition of OSA, did not provide AHI cut-off details or did not provide a prevalence estimate. Where multiple publications arose from a single study, the primary research paper with the largest sample size was chosen for inclusion.

### Quality assessment

Four reviewers (MLG, AJ, AB, AD) independently judged the eligibility of all studies. The risk of bias was systematically evaluated in each eligible study using the Joanna Briggs Institute (JBI) Prevalence Critical Appraisal Tool [22] . This tool is a ten-item questionnaire with four possible responses: yes, no, unclear or not applicable, that assesses limitations related to study design and study population, proportion lost to follow-up, degree of adequate compliance, adjustment for potential confounding factors, and possible conflicts of interest. All articles were reviewed by at least two authors (MLG & AJ). Disagreements between reviewers were resolved by consensus.

### Meta-analysis

Data synthesis was conducted using meta-analysis of proportions via the *metaprop* command in Stata SE 14.2 ^23^ and reported in tables and Forest plots. The random-effects model was chosen *apriori*, since it takes into account differences in study population, and study designs [23] which are assumed to be similar in a fixed methods model. The heterogeneity between studies was assessed using I^2^ statistic with values 0–30%, more than 30 to 60% and more than 60% corresponding to low, moderate and high degree of heterogeneity [24]. To explore potential sources of heterogeneity, sensitivity and subgroup analyses of the prevalence were carried out. Sensitivity analyses were conducted to examine the effect of study quality, by using a method similar to that employed by Patsopoulos and colleagues [25] which involves comparing the pooled prevalence before and after elimination of studies with a rating less than 7/10. Subgroup analysis was conducted where there were sufficient numbers and were categorised by primary OSA assessment technique (PSG versus portable monitor), time of assessment (in hospital versus at least 2 months post-hospitalisation) and assessment during CR program attendance A *p* value of < 0.05 indicated significant heterogeneity between studies [26]. Meta-regression was performed to determine whether possible covariates such as the timing of assessment, sample size, mean age, proportion of women, and study quality could explain the heterogeneity between studies. To assess publication bias among the studies included in the meta-analysis, asymmetry of funnel plots was assessed using Begg’s rank correlation analysis and linear regression analysis (Egger’s method). Since prevalence of OSA during CR was of particular interest a subset of studies that included prevalence estimates at any time during CR was identified for further analysis.

## Results

### Characteristics of the included studies

**Fig. 1:**
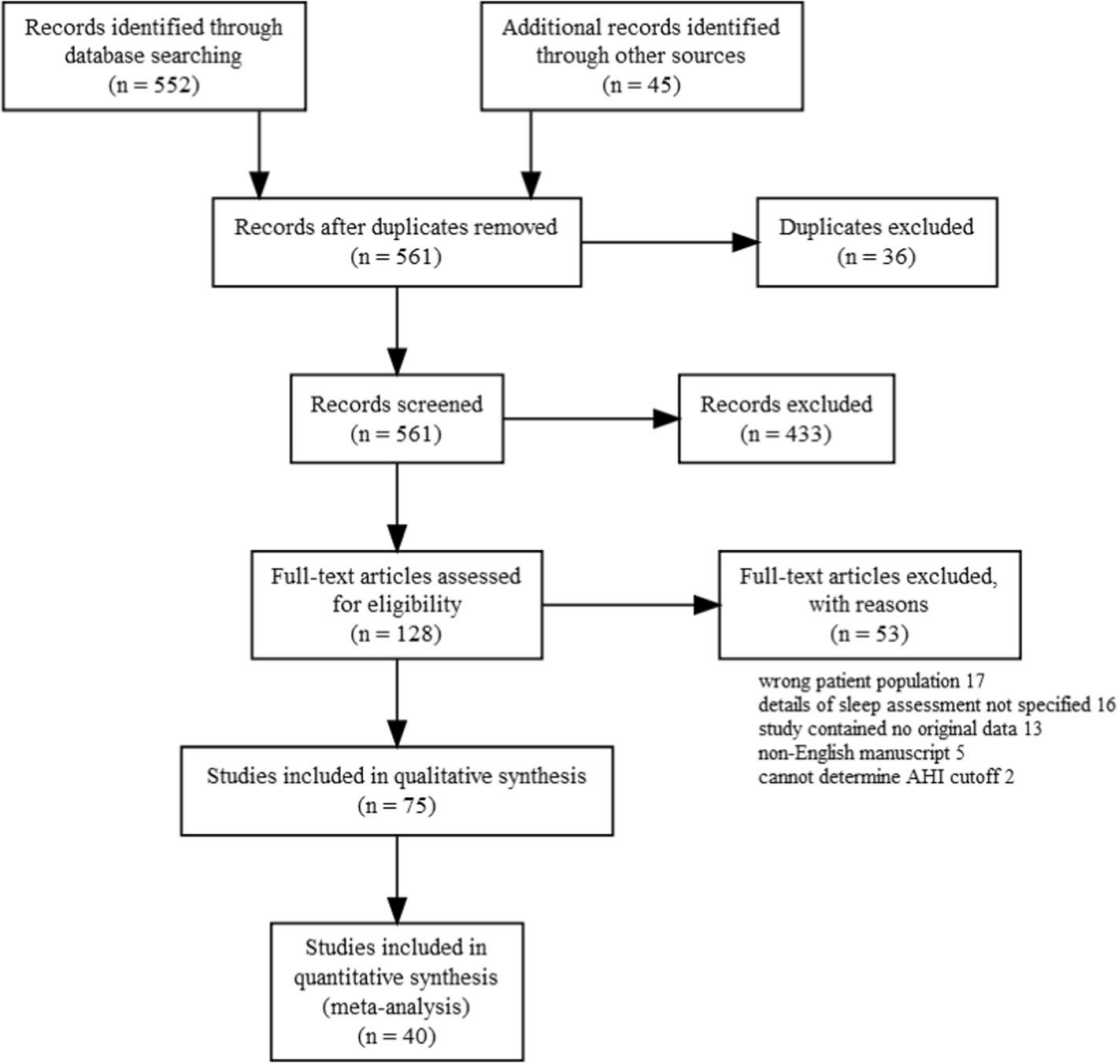
Process of identifying studies for inclusion in the review and meta-analysis

A total of 552 articles were retrieved by literature search (Fig. 1) Of these 128 were screened and 75 retained for full-text evaluation. Finally, after a detailed full-text evaluation using the JBI Prevalence Critical Appraisal Tool (see additional file 3) a total of 40 articles published between 1997 and May 2019 were included in the meta-analysis.

Summary details of the included studies are shown in Table 1 with further details of studies that provided prevalence estimates in Additional file 4. Countries from all regions of the world were represented, with most studies located in North America, South East Asia and Europe. The majority of studies were observational prospective cohort or case-control designs. Approximately two thirds of the selected studies used portable monitoring rather than polysomnography and a number of these assessed more than one level of OSA severity. Assessment was most commonly conducted within the first week of hospitalisation and females represented just one-fifth (20.6%) of all patients.

**Table 1:** Characteristics of studies included for the prevalence estimates

	Apnoea-hypopnoea index (AHI)		
	Mild OSA (AHI ≥5)	Moderate to severe OSA (AHI ≥15)	Severe OSA AHI ≥30)
*Assessment method*			
Polysomnography	7 (44%)	10 (34%)	3 (33%)
Portable monitor	9 (56%)	19 (66%)	6 (67%)
*Timing of assessment (after hospital admission)*			
Pre-operation	0 (0%)	2 (7%)	0 (0%)
< 1 week	8 (50%)	13 (43%)	6 (67%)
1–3 weeks	4 (25%)	9 (30%)	1 (11%)
> 3 weeks	4 (25%)	6(20%)	2 (22%)
*Gender*			
Female n cases	616 (26.9%)	1567 (18.5%)	287 (21.1%)

***OSA*Obstructive sleep apnoea,*AHI*Apnoea/hypoxia index**

### OSA prevalence estimates

**Fig. 2:**
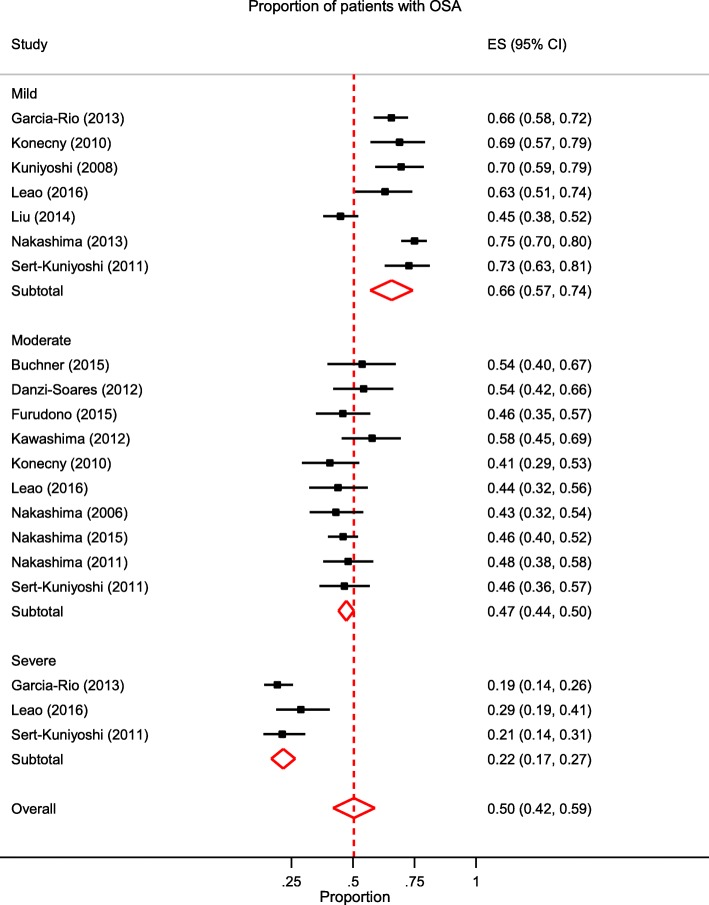
Forest plot of OSA prevalence in studies using polysomnography by OSA severity sub-groups (Mild AHI =5–14; Moderate AHI = 15–29; Severe AHI > 30) post-surgical intervention. ES = effect size; OSA = obstructive sleep apnoea; AHI = apnoea/hypoxia index. See Additional File 4 for references

**Fig. 3:**
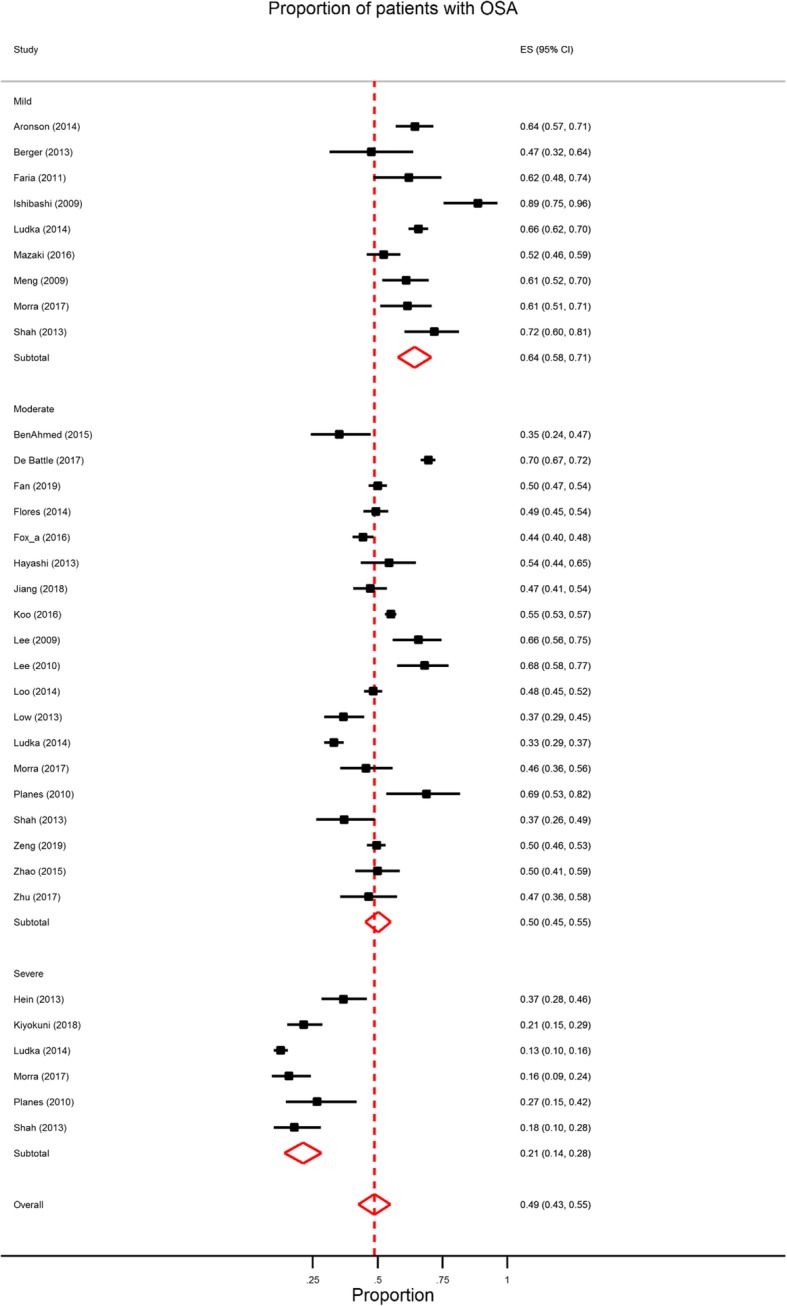
Forest plot of OSA prevalence in studies using portable monitoring by OSA severity sub-groups (Mild AHI =5–14; Moderate AHI = 15–29; Severe AHI > 30) post-surgical intervention. ES = effect size; OSA = obstructive sleep apnoea; AHI = apnoea/hypoxia index. See Additional File 4 for references

The meta-analysis calculated the pooled prevalence of OSA mild (AHI > 5), moderate-to-severe (AHI > 15), and severe (AHI > 30) in ACS patients assessed by either polysomnography (Fig. 2) or portable home monitoring (Fig. 3) following surgical intervention. Similar pooled prevalence estimates for OSA were obtained for both methods at the moderate (close to half of all patients for both) and severe cut-off levels (over a fifth of all patients) (see Table 2). Study heterogeneity was acceptable for the polysomnography estimates but was moderate to high for portable monitoring.

**Table 2:** Prevalence estimates as a function of OSA severity and method of assessment

	No. studies	Random pooled prevalence (95% CI)	Significance tests of prevalence = 0	Heterogeneity Statistic (Q)	I^2^a^	Sensitivity prevalence^b^	Rank correlation test (Begg)	Reg test for funnel plot asymmetry (Egger)
**Mild OSA**								
**AHI ≥ 5**								
PSG	7	0.66 (0.57–0.74)	z = 14.91, *p* < 0.001	51.24, *p* < 0.001	88.29%	0.66 (0.57–0.74)	0.23, *p* = 0.517	−1.5, *p* = 0.125
Portable monitor	9	0.63 (0.57–0.69)	z = 19.13, *p* < 0.001	46.18, *p* < 0.001	82.68%	0.63 (0.57–0.68)	−0.16, *p* = 0.612	−0.6, *p* = 0.566
**Moderate OSA**								
**AHI ≥ 15**								
PSG	10	0.47 (0.44–0.50)	z = 29.78, *p* < 0.001	8.16, *p* = 0.520	0.01%	0.47 (0.43–0.50)	0.16, *p* = 0.601	0.6, *p* = 0.529
Portable monitor	19	0.50(0.45–0.55)	z = 19.27, *p* < 0.001	81.13, *p* < 0.001	94.59%	0.49 (0.44–0.55)	0.02, *p* = 0.965	0.1, *p* = 0.925
**Severe OSA**								
**AHI ≥ 30**								
PSG	3	0.22 (0.17–0.27)	z = 8.74, *p* < 0.001	2.49, *p* = 0.287	22.70%	0.22 (0.17–0.27)	0.98, *p* = 0.333	1.5, *p* = 0.141
Portable monitor	6	0.21 (0.14–0.28)	z = 5.82, *p* < 0.001	34.77, *p* < 0.001	85.62%	0.21 (0.13–0.28)	0.14, *p* = 0.103	1.98. *p* = 0.080

^a^ I^2: the variation in ES attributable to heterogeneity; *PSG* Polysomnography, *OSA* Obstructive sleep apnoea, *AHI* Apnoea/hypoxia index

^b^ Sensitivity analysis: removal of 8 studies with quality rating below 7/10

### Sensitivity analyses

**Fig. 4:**
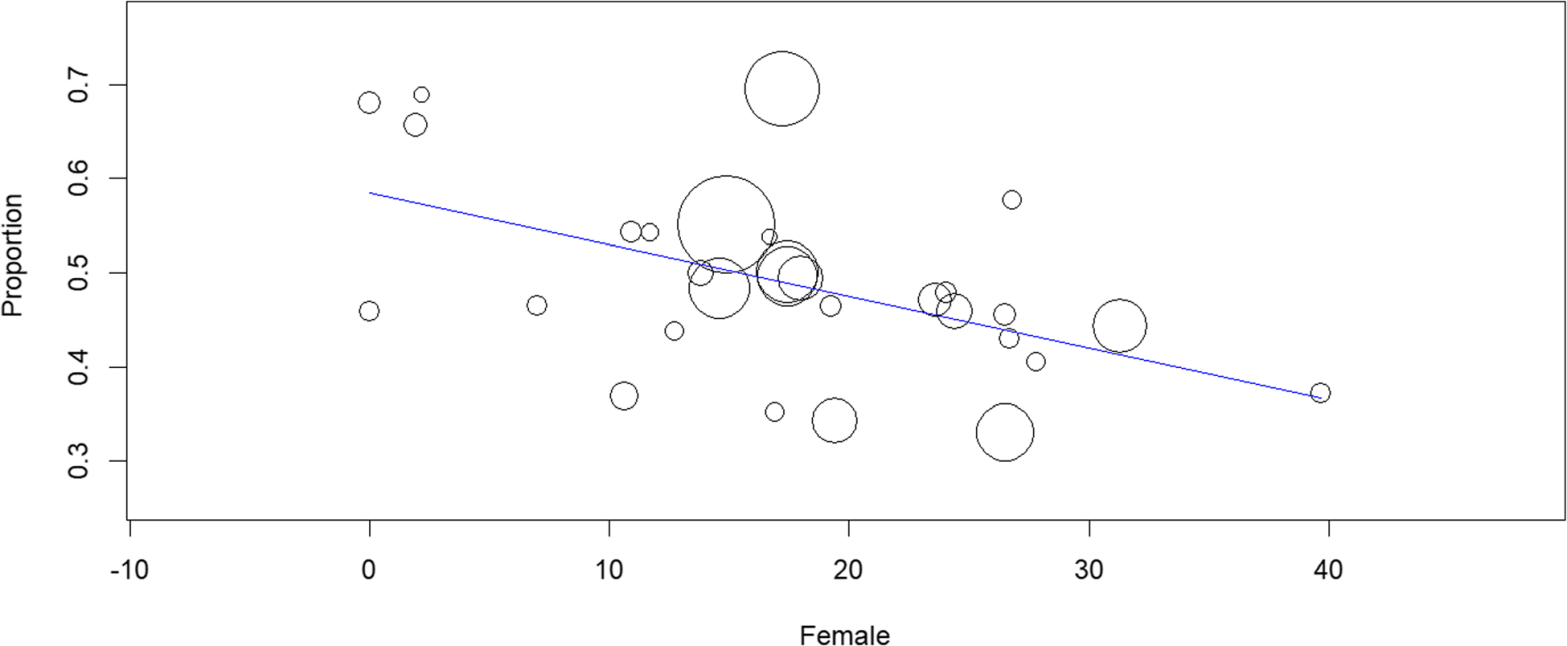
Meta-regression of percentage female included in each study and effect on moderate OSA (AHI 15–29) prevalence estimate. **OSA = obstructive sleep apnoea; AHI = apnoea/hypoxia index**

The sensitivity analysis revealed no significant changes to the prevalence estimates when lower quality studies were removed (Table 2). Further, a meta-regression indicated that JBI quality of study rating did not significantly influence prevalence estimates (β = − 0.007 (95% CI − 0.038 to 0.024), *p* = 0.650). The effect of timing of OSA assessment was also examined (Table 3). The prevalence of mild OSA using polysomnography within 2 weeks of the ACS event, was 5% lower than at greater than 2 weeks of the OSA event. Using portable monitoring, a significant rise (13%) in mild OSA prevalence was observed over time. In contrast, for the moderate OSA estimates, there were decreases in the prevalence estimate over time, 8% using polysomnography and 1% using portable monitoring. The severe OSA estimates increased by 2%. A regression analysis on the effect of timing on the moderate OSA prevalence estimate using portable monitoring revealed a negative slope (β = − 0.05) with estimates declining over time but this was not significant (*p* = 0.851). Using the data restricted to moderate OSA, a meta-regression was also performed using percentage of female participants in each study. There was a significant negative slope β = − 0.05 (95% CI − 0.009 to − 0.002), *p* = 0.002) indicating that prevalence estimates decreased as percentage of females increased (see Fig. 4).

**Table 3:** Effect of time of assessment after surgical procedure on prevalence estimate

*Time after procedure*	No. studies	Random pooled prevalence (95% CI)	Significance tests of prevalence = 0	Heterogeneity statistic	df	*P*	I^2*
**Mild OSA AHI ≥ 5**							
*PSG*							
Within 2 weeks	2	0.67 (0.61–0.72)	z = 24.01, p = 0.00	–	–	–	–
> 2 weeks	4	0.72 (0.67–0.76)	z = 28.72, *p* = 0.00	4.22	3	0.24	28.94%
Test for heterogeneity between timing sub-groups 1.51, df = 1, *p* = 0.22							
*Portable*							
Within 2 weeks	6	0.59 (0.53–0.65)	z = 19.80, *p* = 0.00	16.24	5	0.01	69.22%
> 2 weeks	3	0.72 (0.55–0.89)	z = 8.43, *p* = 0.00	–	–	–	–
Test for heterogeneity between timing sub-groups 2.01, df = 1, *p* = 0.16							
**Moderate OSA AHI ≥ 15**							
*PSG*							
Within 2 weeks	4	0.51 (0.45–0.57)	z = 16.75, *p* = 0.00	4.37	3	0.22	31.28%
> 2 weeks	6	0.43 (0.39–0.48)	z = 19.98, *p* = 0.00	4.40	6	0.49	0.01%
*Portable*							
Within 2 weeks	16	0.50 (0.44–0.56)	z = 16.84, *p* = 0.00	36.77	15	0.00	95.26%
> 2 weeks	3	0.49 (0.43–0.54)	z = 18.73, *p* = 0.00	–	–	–	–
Test for heterogeneity between timing sub-groups 0.20, df = 1, *p* = 0.65							
**Severe OSA AHI ≥ 30**							
*PSG & Portable combined*							
Within 2 weeks	8	0.22 (0.15–0.29)	z = 6.47, *p* = 0.00	39.26	7	0.00	84.72%
> 2 weeks	2	0.24 (0.18–0.30)	z = 7.41, *p* = 0.00	–	–	–	–
Test for heterogeneity between timing sub-groups 0.16, df = 1, *p* = 0.69							

^a^ I^2: the variation in ES attributable to heterogeneity; *PSG* Polysomnography, *OSA* Obstructive sleep apnoea, *AHI* Apnoea/hypoxia index

### Publication bias

No significant publication bias was detected using either Begg’s or Egger’s methods for assessing funnel plot asymmetry (Table 2). method. Further, visual inspection of the funnel plots indicated that they were symmetrical.

### Probability of OSA upon entry to CR programs

Four studies had assessed the probability of an OSA diagnosis associated with attendance at CR programs, but only two had made the assessments upon entry to CR (Table 4). Estimates of those at risk of OSA upon CR entry ranged from 53 to 65% of participants. When portable monitoring was utilised estimates of mild OSA ranged from 44 to 66%, and moderate to high OSA ranged from 33 to 75%. The one study that utilised polysomnography [27] found that close to three-quarters of participants had a diagnosis of mild OSA upon entry to the CR program. In accordance with the overall PSG and portable monitoring prevalence estimates for all ACS patients, the study using Holter ECG monitoring [28] classified 50% of the CR patients as having at least moderate to severe OSA before entry into CR (see Table 4).

**Table 4:** Prevalence of self-reported OSA in CR settings.

Author (year)	Method of assessing OSA risk	Numbers with OSA/total N	Timing of assessment	Prevalence Estimate
***Questionnaire probability of OSA***				
Sharma & Parker,2011 [47]	Berlin Questionnaire	63/118	Upon entry to CR	53% at risk of OSA
Sert-Kuniyoshi et al. 2011 [27]	Berlin Questionnaire	64/99	Upon entry to CR	65% at risk of OSA
Marzolini et al. 2016 [46]	STOP-Bang	174/211	At any time in the CR program	82% at risk of OSA
Loo et al. 2016 [52]	Berlin Questionnaire STOP-Bang	123/332 177/332	Late phase CR	37% at risk of OSA 53% at risk of OSA
***Objective monitoring***				
Fox et al. 2016 [44]	Apnealink	264/595	Upon entry to CR	AHI ≥ 5 44%
Hargens et al. 2015 [53]	Apnealink	47/73	Upon entry to CR	AHI ≥ 5 66%
Skobel et al. 2015 [54]	Apnealink	380/1152	First week after entry to CR	AHI ≥15 33%
Loo et al. 2016 [52]	Watch- PAT	69/209	Late phase CR	AHI ≥15 33%
Sert-Kuniyoshi et al. 2011 [27]	Overnight PSG	72/99	Upon completion of CR program	AHI ≥5 73%
Hupin et al. 2018 [28]	Overnight Holter ECG	67/105 52/105 37/105	7–21 days post MI before entry to CR	AHI ≥5 64% AHI ≥15 50% AHI ≥30 35%

*CR* Cardiac rehabilitation, *PSG* Polysomnography, *ECG* Electrocardiogram, *PAT* Peripheral arterial tone, *OSA* Obstructive sleep apnoea, *AHI* Apnoea/hypoxia index.

## Discussion

This meta-analysis confirmed a high prevalence of OSA in ACS patients that persisted within the first six weeks following hospital admission. Close to two-thirds of ACS patients exhibited mild to moderate OSA. What are the mechanisms that could potentially explain this high prevalence level in ACS patients? It is though that the prominent features of OSA, intermittent hypoxia and sleep fragmentation, could affect a number of pathophysiological pathways including metabolic dysfunction [29], sympathetic activation [30], inflammation [31] and oxidative stress [32]. It is known that even mild levels of OSA with minimal daytime symptoms are associated with adverse effects on endothelial function and hypertension [33, 34]. Importantly, higher risk of recurrent ACS has been found at this mild AHI cut-off level compared to patients with no symptoms of OSA [35]. Alarmingly, close to half of all patients were diagnosed with moderate to severe OSA, a level that has been independently associated with 2.3 times the risk of recurrent ACS events after adjusting for covariates such as age, sex, BMI, smoking and diabetes [36].

A second major finding from this study concerns the method of diagnosis of OSA. Remarkably similar estimates of prevalence were obtained using level I “gold standard” polysomnography and levels II to IV portable monitoring, particularly at the moderate and severe AHI levels. A recent review of OSA assessment in CVD patients reported considerable intra-individual night-to-night variability in AHI assessed by polysomnography, with variability particularly higher in patients with mild OSA [37]. These findings would imply that longer periods of assessment, certainly greater than just one night, are warranted in order to gain a more accurate diagnosis of OSA or days of burden with OSA. Given the high congruence in prevalence estimates obtained in this study, the night to night variability of AHI, and the considerable expense associated with polysomnography over a number of nights, it may therefore be of more practical benefit to utilise those portable monitoring devices which have been found to have good predictive validity [38] in long term OSA assessment of ACS patients.

Given the high OSA prevalence estimates obtained in this study it is important to consider the potential implications for screening of this condition upon entry to CR programs. A recent systematic review of exercise-based CR programs reported a null effect of such programs on mortality outcomes [39]. In response to this review it has been pointed out that exercise dose and adherence to exercise may have varied significantly across individual trials, a factor that could explain the heterogeneity in exercise capacity observed across trials and partially explain the overall null finding [40]. The high prevalence of OSA observed in patients attending CR, as noted in this study (Table 4), would contribute to this heterogeneity of exercise adherence and exercise capacity [7, 8] and undermine the effectiveness of exercise-based CR programs. In addition, recent meta-analyses have indicated significant reductions in cardiovascular morbidity [41] and mortality [42] following continuous positive airway pressure (CPAP) treatment where adequate adherence was observed. Given the increased risk for recurrent ACS and the potentially reduced effectiveness of exercise-based CR treatment it would make sense to screen for this condition upon entry to out-patient cardiac rehabilitation programs [19].

There are several potential limitations of this review that should be addressed. First, the vast majority of studies selected in this review included studies based in hospital coronary care units. It was not possible to perform a sub-group analysis on patients attending CR which is typically 4–8 weeks after hospital discharge. In keeping with previous findings [16, 17], our meta-regression findings indicated that OSA prevalence estimates tend to decline slightly in the weeks following hospital admission, however this is not a significant decrease. Individual studies that utilised objective measurement [27, 43, 44] and questionnaires [45–47] support the conclusion that OSA prevalence is high upon entry to CR programs. Second, our meta-regression confirmed that any estimate of OSA prevalence is influenced by gender mix, with lower prevalence rates obtained when greater percentages of females are included in the study. The majority of studies selected in this review include predominantly male patients and there were not enough studies to conduct a meta-analysis where equal gender balance was reported. It is known that women are less likely to be referred for revascularization for ACS than men [48], so it is likely that this issue will persist in future studies. Third, due to lack of specific reporting of OSA by diagnostic category in the majority of studies, we were not able to perform sub-group analysis comparing levels of severity of ACS severity. Limited evidence does suggest that severity of OSA may be marginally higher in CABGS patients than PCI patients [49, 50], however this is confounded by insufficient testing in patients who perceive themselves to be asymptomatic and do not participate in studies [49].

Finally, it is important to acknowledge the considerable heterogeneity across the studies included, particularly among studies that utilised portable monitoring. Efforts to account for this heterogeneity including sub-group analysis could not effectively reduce heterogeneity statistics obtained. Given the large variety of type II through to type IV portable devices utilised with varied number of channels recorded [51], varying predictive validity [38], used by different health professionals and the variety of conditions in which they were utilised (e.g. at home, in hospital, expert assisted or not) it may not be possible to effectively control for this study heterogeneity. It is important to note that acceptable heterogeneity was observed for polysomnography where similar prevalence estimates were obtained. Finally, the traumatisation of hospitalisation and surgical intervention is often associated with sleep disturbances which can include OSA and insomnia.

## Conclusions

OSA was found to be highly prevalent in ACS patients immediately following surgical intervention and remains high in the period where entry to CR programs is typical. Given the evidence that OSA has a deleterious impact upon exercise capacity and adherence and adversely affect CR efforts,, but can be effectively treated, it may be advisable to screen for this condition upon CR program entry.

## Supplementary information

**Additional file 1.** PRISMA-P (Preferred Reporting Items for Systematic review and Meta-Analysis Protocols) 2015 checklist: recommended items addressed in our systematic review and meta-analysis.

**Additional file 2.** Search strategy.

**Additional file 3.** Quality ratings for prevalence studies.

**Additional file 4.** Details of publications providing a prevalence estimate.

## Data Availability

The datasets supporting the conclusions of the study are included in the article. Any additional data will be available on request. Michael Le Grande should be contacted to request the data.

## Abbreviations

OSA: Obstructive sleep apnoea
ACS: Acute coronary syndrome
CHD: Coronary heart disease
CR: Cardiac rehabilitation
AHI: Apnoea-hypoxia index
PSG: Polysomnography
PCI: Percutaneous coronary intervention
CABGS: Coronary artery bypass graft surgery
BMI: Body mass index
