# Supplementary material for: Prevalence of obstructive sleep apnoea in acute coronary syndrome patients: systematic review and meta-analysis
Source: BMC Cardiovasc Disord. 2020 Mar 24;20:147. doi: 10.1186/s12872-020-01430-3 (PMC7092582; doi:10.1186/s12872-020-01430-3)
Supplement: Supplementary file 2 — Additional file 2. Search strategy. [file 12872_2020_1430_MOESM2_ESM.docx]

Additional file 2

| **Database** | **Search Strategy** |
| --- | --- |
| **MEDLINE via PubMed** | ("obstructive sleep apnea"[tw] OR "obstructive sleep apnoea"[tw] OR "sleep disordered breathing"[tw]) AND ("acute coronary syndrome"[All Fields] OR "myocardial infarction"[All Fields] OR "unstable angina"[All Fields]) |
| **EMBASE** | #1 'acute coronary syndrome'  #2 'sleep disordered breathing'  #3 #1 AND #2 AND [humans]/lim AND [abstracts]/lim |
| **CINAHL** | (sleep apnea OR sleep apnoea OR sleep disordered breathing)  AND (acute coronary syndrome OR myocardial infarction OR unstable angina) |
| **Google Scholar** | (“obstructive sleep apnea” OR “obstructive sleep apnoea” OR “sleep disordered breathing”)  AND (“acute coronary syndrome” OR “myocardial infarction” OR “unstable angina”) |
| **Cochrane Library** | #1 MeSH descriptor: [Sleep Apnea, Obstructive] explode all trees  #2 MeSH descriptor: [Acute Coronary Syndrome] explode all trees  #1 AND #2 |
