# Supplementary material for: Prevalence of obstructive sleep apnoea in acute coronary syndrome patients: systematic review and meta-analysis
Source: BMC Cardiovasc Disord. 2020 Mar 24;20:147. doi: 10.1186/s12872-020-01430-3 (PMC7092582; doi:10.1186/s12872-020-01430-3)
Supplement: Supplementary file 3 — Additional file 3. Quality ratings for prevalence studies. [file 12872_2020_1430_MOESM3_ESM.docx]

| **Study author and year** | **1. Was the sample representative of the target population?** | **2. Were study participants recruited in an appropriate way?** | **3. Was the sample size adequate?** | **4. Were the study subjects and setting described in detail?** | **5. Was the data analysis conducted with sufficient coverage of the identified sample?** | **6. Were valid methods used for the identification of the condition?** | **7. Was the condition measured in a standard, reliable way for all participants?** | **8. Was there appropriate statistical analysis?** | **9. Was the response rate adequate, and if not, was the low response rate managed appropriately?** | **Total number of “yes”** | **Include or Exclude in meta-analysis** |
| --- | --- | --- | --- | --- | --- | --- | --- | --- | --- | --- | --- |
| Aronson 2014 | u | y | y | y | y | y | y | y | y | 8 | y |
| BenAhmed 2015 | y | y | n | y | y | y | y | y | n | 7 | y |
| Berger 2013 | y | y | n | y | y | u | y | y | u | 6 | y |
| Buchner 2015 | y | y | n | y | y | y | y | y | y | 8 | y |
| Danzi-Soares 2012 | y | y | n | y | y | y | y | y | y | 8 | y |
| De Battle 2017 | y | y | y | y | y | y | y | y | y | 9 | y |
| Fan 2019 | y | y | y | y | y | y | y | y | y | 9 | y |
| Faria 2011 | y | y | y | u | y | y | y | u | u | 6 | y |
| Florés 2014 | y | u | y | n | y | y | y | y | u | 6 | y |
| Fox 2016 | y | y | y | y | y | y | y | y | y | 9 | y |
| Furudono 2015 | u | u | y | n | y | y | y | y | u | 5 | y |
| Garcia-Rio 2013 | y | y | y | y | y | y | y | y | y | 9 | y |
| Hayashi 2013 | y | y | y | y | y | y | y | y | u | 8 | y |
| Hein 2013 | y | u | y | y | y | y | y | y | u | 7 | y |
| Ishibashi 2009 | y | u | n | y | y | y | y | y | u | 6 | y |
| Jiang 2018 | y | y | y | n | y | y | y | y | u | 7 | y |
| Kawashima 2012 | y | u | y | u | y | u | y | y | u | 5 | y |
| Kiyokuni 2018 | y | y | y | y | n | y | y | y | u | 7 | y |
| Konecny 2010 | y | y | y | y | y | y | y | y | u | 8 | y |
| Koo 2016 | y | y | y | y | y | y | y | y | u | 8 | y |
| Kuniyoshi 2008 | y | y | y | y | y | y | y | y | u | 8 | y |
| Leão 2016 | y | y | n | y | y | y | y | y | u | 7 | y |
| Lee 2009 | n | y | y | y | n | y | y | y | u | 6 | y |
| Lee 2010 | u | y | y | y | y | y | y | y | u | 7 | y |
| Liu 2014 | y | u | y | y | y | y | y | y | u | 7 | y |
| Loo 2014ª | y | y | y | y | y | y | y | y | y | 9 | y |
| Low 2013 | y | y | y | y | y | y | y | y | u | 8 | y |
| Ludka 2014 | y | y | y | y | y | y | y | y | y | 9 | y |
| Mazaki 2016 | y | y | y | n | y | y | y | y | u | 7 | y |
| Meng 2009 | y | y | y | y | y | y | y | y | u | 8 | y |
| Morra 2017 | y | y | y | y | y | y | y | y | u | 8 | y |
| Nakashima 2006 | y | y | n | y | y | y | y | y | u | 7 | y |
| Nakashima 2015 | y | y | y | y | y | y | y | y | u | 8 | y |
| Nakashima 2011 | y | y | n | y | y | y | y | y | u | 7 | y |
| Planes 2010 | y | y | n | n | y | y | y | y | n | 6 | y |
| Sert-Kuniyoshi 2011 | y | y | n | y | y | y | y | y | y | 8 | y |
| Shah 2013 | y | y | y | y | y | y | y | y | u | 8 | y |
| Zeng 2019 | y | y | y | y | y | y | y | y | u | 9 | y |
| Zhao 2015b | y | y | n | y | y | y | y | y | u | 7 | y |
| Zhu 2017 | y | y | y | y | y | y | y | y | u | 9 | y |

Reference list is provided in Additional file 4
