# Supplementary material for: Prevalence of obstructive sleep apnoea in acute coronary syndrome patients: systematic review and meta-analysis
Source: BMC Cardiovasc Disord. 2020 Mar 24;20:147. doi: 10.1186/s12872-020-01430-3 (PMC7092582; doi:10.1186/s12872-020-01430-3)
Supplement: Supplementary file 4 — Additional file 4. Details of publications providing a prevalence estimate. [file 12872_2020_1430_MOESM4_ESM.docx]

**References providing a prevalence estimate**

| Polysomnography  AHI 5 | | | | | | | | | |
| --- | --- | --- | --- | --- | --- | --- | --- | --- | --- |
| Study author and year | **Country/No. enrolled/setting** | **Diagnosis** | **Gender** | **Age (mean)** | **Timing** | **Method** | **No with OSA** | **Total N tested** | **% with OSA** |
| Garcia-Rio 2013^1^ | Spain/CCU | AMI | 166 (86.5%) M  26 (13.5%) F | Mean age 58 | on days 1–3  after hospital admittance | Prospective case-control observational study.18-channel overnight polysomnography | 126 | 192 | 65.6% |
| Konecny 2010 ^2^ | USA/798 patients  admitted to our institution with the diagnosis of acute MI, 74 with suspected OSA chosen for PSG | AMI | 46 (62.2%) M  28 (37.8%) F | Mean age 62 | within 6 weeks of the MI hospitalization | Prospective case-control observational study. Overnight polysomnography. / No ESS | 51 | 74 | 69% |
| Kuniyoshi 2008^3^ | USA/ Mayo Clinic We prospectively studied 92 patients admitted with incident MI | AMI | 71 (77.2%) M  21 (22.8%) F | Mean age 61 | 17 ⫾ 2.4 days after MI | Prospective case-control observational study. Overnight polysomnography/ No ESS | 64 | 92 | 70% |
| Leão 2016^4^ | Portugal/73 patients admitted on cardiac intensive care unit for ACS. | ACS | 55 (87.3%) M  18 (12.7%) F | Mean age 62.4 | after clinical stabilization 55 days median | Prospective case-control observational study. PSG was performed with the oversight of a sleep technologist using an Alice 4 device./ ESS included | 46 | 73 | 63% |
| Liu 2014^5^ | China/ 198 patients diagnosed as STEMI | AMI | 132 (66.7%) M 66 (33.3%) F | Mean age 61.5 | During hospital stay in first week | Retrospective cohort study. Overnight polysomnography/No ESS | 89 | 198 | 44.9% |
| Nakashima 2013^6^ | Japan/ Hospital | AMI | 220 (76.4%) M  68 (23.6%) F | Mean age 66 | between 14 and 21 days. | Prospective case-control observational study. Overnight polysomnography | 216 | 288 | 75% |
| Sert-Kuniyoshi 2011^7^ | USA/ Mayo clinic | MI | 80 (80.8%) M 19 (19.2%) F | Mean age 62 | 1 to 3 months after MI | Prospective case-control observational study. Comprehensive, digital, full-night diagnostic PSG/BQ/ESS | 72 | 99 | 73% |
| AHI 15 | | | | | | | | | |
| Buchner 2015^8^ | Germany/ 74 first-time AMI (ST elevation on ECG or complete occlusion of the coronary artery in non-ST elevation MI and PCI | AMI | 45 (83.3%) M 9 (16.7%) F | 55 SDB  53 no SDB | 3-5 days after PCI  And 12 weeks | Prospective case-control observational study. Overnight PSG | 29 | 54 (20 excluded) | 54% 40% 12w |
| Danzi-Soares 2012^9^ | Brazil | CABGS | 53 (88.3%) M 17 (11.7%) F | Mean age 58 | In the preoperative period 2–3 days before surgery | Prospective case-control observational study. Polysmonography (XactTrace, EMBLA Medical Devices, Broomfield, Colorado, USA)/ portable sleep monitor/BQ/ESS | 38 | 70 | 54.3% |
| Furudono 2015^10^ | Japan/ 85 patients with AMI who underwent PCI | AMI | NA | NA | Day 14 after AMI | Prospective case-control observational study. Polysomnography | 39 | 85 | 45.9% |
| Kawashima 2012^11^ | Japan/71 consecutive patients with ACS who were treated by PCI within 24 hours after the admission | ACS | 52 (73.2%) M  19 (26.8%) F | Mean age 68 | Within 24 hours of admission | Prospective case-control observational study “sleep study” ?/No ESS | 41 | 71 | 57.7% |
| Konecny 2010^2^ | USA/798 patients  admitted to our institution with the diagnosis of acute MI, 74 with suspected OSA chosen for PSG | AMI | 46 (62.2%) M  28 (27.8%) F | Mean age 62 | within 6 weeks of the MI hospitalization | Prospective case-control observational study. Overnight polysomnography/ No ESS | >15 30 | 74 | >15 41% |
| Leão 2016^4^ | Portugal/73 patients admitted on cardiac intensive care unit for ACS. | ACS | 55 (87.3%) M  18 (12.7%) F | Mean age 62.4 | after clinical stabilization 55 days median | Prospective case-control observational study. PSG using an Alice 4 device./ ESS included | 25 | 73 | 34% |
| Nakashima 2006^12^ | Japan/ Hospital | AMI | 63 (73.3%) M 23 (26.7%) F | Mean age 68 OSA/65 non-OSA | between 14 and 21 days. | Prospective case-control observational study. polysomnography | 37 | 86 | 43.0% |
| Nakashima 2015^13^ | Japan/ Hospital | AMI | 204 (75.6%) M  66 (24.4%) F | Mean age 71 OSA/65 non-OSA | Before hospital discharge | Prospective case-control observational study. 18-channel polysomnography | 124 | 270 | 45.9% |
| Nakashima 2011^14^ | Japan/ Hospital | AMI | 76 (76%) M 24 (24%) F | Mean age 66 OSA/64 non-OSA | between 14 and 21 days. | Prospective case-control observational study. polysomnography | 48 | 100 | 48% |
| Sert-Kuniyoshi 2011^7^ | USA/ Mayo clinic | MI | 80 (80.8%) M 19 (19.2%) F | Mean age 62 | 1 to 3 months after MI | Prospective case-control observational study. comprehensive, digital, full-night diagnostic PSG/BQ/ESS | 46 | 99 | 46% |
| AHI 30 | | | | | | | | | |
| Garcia-Rio 2013^1^ | Spain/CCU | AMI | 166 (86.5%)M  26 (13.5%) F | Mean age 58 | on days 1–3  after hospital admittance | Prospective case-control observational study. Overnight polysomnography 18-channel polysomnograph | 126 | 192 | ≥30 19.3 |
| Leão 2016^4^ | Portugal/73 patients admitted on cardiac intensive care unit for ACS. | ACS | 55 (87.3%) M  18 (12.7%) F | Mean age 62.4 | after clinical stabilization 55 days median | Prospective case-control observational study. PSG was performed with the oversight of a sleep technologist using an Alice 4 device/ ESS included | 21 | 73 | 29% |
| SertKuniyoshi 2011^7^ | USA/ Mayo clinic | MI | 80 (80.8%) M 19 (19.2%) F | Mean age 62 | 1 to 3 months after MI | Prospective case-control observational study comprehensive, digital, full-night diagnostic PSG/BQ/ESS | 21 | 99 | 30 21% |

| Portable home monitoring  AHI 5 | | | | | | | | | |
| --- | --- | --- | --- | --- | --- | --- | --- | --- | --- |
| Study author and year | **Country/No. enrolled/setting** | **Diagnosis** | **Sex** | **Age** | **Timing** | **Method** | **No with OSA** | **Total N tested** | **% with OSA** |
| Aronson 2014^15^ | Israel/220 AMI patients who had survived the first 5 days | AMI | 150 (83.3%)M 30 (16.7%)F | 56 – no SDB  59 - SDB | several days after event (preferably last day of hospital admission) | Prospective case-control observational study .Watch-Pat-100 sleep study | 116 | 180 (40 excluded for technical issues) | 64% |
| Berger 2013^16^ | 40 | AMI | 40 (100%) M  0 (0%) F | Mean age 57 | Most within one day of admission | Prospective case-control observational study. WatchPAT-100 | 19 | 40 | 47.5% |
| Faria 2011^17^ | Italy/ patients consecutively admitted to CCU with ACS | ACS | 43 (74.1%) M  15 (25.9%) F | Mean 62 | Median 17.5 days | Prospective case-control observational study. Apnea Link device | 36 | 58 | 62% |
| Ishibashi 2009^18^ | Japan/Hospital | ACS | 36 (81.8%) M  8 (18.2%) F | Mean age 60.6 | 2—3 weeks after the onset of ACS | Prospective case-control observational study. A portable PSG (Morpheus R®, Teijin Limited, Tokyo, Japan)/No ESS | 39 | 44 | 88.6% |
| Ludka 2014^19^ | Czech Republic/ 2 hospitals | AMI | 446 (73.5%) M  161 (26.5%) F | Mean age 62.0 | median duration from hospital admission to overnight sleep study was 4 days (range 2 to 14 days) | Prospective case-control observational study. Portable diagnostic device [Apnealink™ (ResMed) | 399 | 607 | 65.7% |
| Mazaki 2016^20^ | Japan/ Hospital | ACS | 185 (76.8%) M  56 (23.2%) F | Mean age 64 OSA/63 non-OSA | Within 1 week after the onset of ACS during hospitalization | Prospective case-control observational study. Portable cardiorespiratory monitoring device (Pulsleep LS100; Fukuda Denshi Co, Ltd) | 126 | 241 | 52.2% |
| Meng 2009^21^ | China/Hospital | ACS | 85 (69.1%) M 38 (30.9%) F | Mean age 66 | Within 7 days after the PCI procedure | Prospective case-control observational study. Unattended portable system (Compumedics Sleep S-Series; Compumedics Ltd, Abbotsford | 75 | 123 | 61% |
| Morra 2017^22^ | France/Coronary care unit | ACS | 74 (73.3%) M 27 (26.7%) F | Mean age 58.7 | Within 1-3 days of admission to ICU | Prospective case-control observational study. Portable ApneaLink ResMed/ESS | 62 | 101 | 61.4% |
| Shah 2013^23^ | USA/ Hospital | MI | 47 (60.3%) M 31 (39.7%) F | Mean age 58 | In hospital lstudied shortly after the onset of acute MI | Prospective case-control observational study. Apnealink Plus monitor type III portable device/ESS/BQ | 56 | 78 | 71.8% |
| AHI 15 | | | | | | | | | |
| BenAhmed 2015^24^ | Israel/119 patients with an inaugural myocardial infarction who had undergone a coronary angiography  within 24 h of onset | AMI | 59 (83.1%) M 12 (16.9%) F | Mean age 59 | underwent an overnight polygraph before discharge | Prospective case-control observational study. Overnight portable diagnostic device (Medibyte Junior 2.0, Braebon, Ontario, Canada) | 25 | 71 (48 excluded) | 35.2% |
| De Battle 2017^25^ | Spain/ISAACC STUDY | ACS | 810 (82.8%) M  168 (17.2%) F | Mean 58 <no OSA, 60 OSA | during the first 48–72 h after admission | Prospective case-control observational study. overnight cardio-respiratory polygraphy (Embletta; ResMed, Bella Vista, Australia) | 680 | 978 | 69.5% |
| Fan 2019^26^ | China | ACS | 664 (82.6%) M 104 (17.4%) F | Mean age 57.5 | During hospitalization (within 2 weeks after admission) | Portable cardiorespiratory polygraphy (ApneaLink, Resmed, Australia) | 403 | 804 | 50.1% |
| Flores^27^ | Spain | ACS | 353 (82.0%) M 78 (18%) F | Mean age 60.5 | In the first 72 h after admission | Polygraphy (Embletta; ResMed, Spain | 213 | 431 | 49.4% |
| Fox 2016^28^ | Germany/Patients attending cardiac rehab | ACS | 409 (68.7%) M  176 (31.3%) F | Mean age 72 | Mean of 38.8 ± 28.2 days between any ACS and PG screening | Prospective case-control observational study. Portable polygraphy (PG) including pulse oximetry (ResMed ApneaLink Oxy | 264 | 595 | 44.4% |
| Hayashi 2013^29^ | Japan/92 consecutive patients who  had been admitted for a first acute MI | AMI | 82 (89.1%) M 10 (10.9%) F | mean age of 65 ± 12 years. | 14 days after the onset of acute MI (acute phase). | Prospective case-control observational study. Sleep monitor (Pulsleep LS-120S, Fukuda Denshi, Tokyo, Japan)/ESS | 50 | 92 | 54.3% |
| Jiang 2018^30^ | China/Hospital/patients who had PCI for ACS | ACS | 185(76.4%) M  57 (23.6%) F | Mean age 64 | After PCI procedure | Embletta Gold standardized level‑3 portable diagnostic system | 114 | 242 | 47.1% |
| Koo 2016^31^ | Spain/Singapore/ 1961 patients from 2 data sets: ISAACC Trial and Sleep and Stent Study | ACS | 1667(85.1%) M 292(14.9%) F | Mean age 57.7 | conducted during the index admission, usually within 24 to 72 hours of admission. | Secondary analysis of 2 studies – a case-control observational study and a randomised controlled trial. Portable cardiopulmonary diagnostic device (Embletta Gold, Natus Medical Inc., Canada) / No ESS | 1080 | 1959 | 55.1% |
| Lee 2009^32^ | Singapore/120 patients admitted to coronary care unit | AMI | 103 (98.1%) M 2 (1.9%) F | Mean age 53 | between 2 and 5 days after primary PCI | portable diagnostic  device (Somte; Compumedics; Melbourne, VIC, Australia)/ No ESS | 69 | 105 | 65.7% |
| Lee 2010^33^ | Singapore/105 patients who were admitted to our institution for first acute ST elevation myocardial infarction | AMI | 94 (100%)M  0 (0%) F | Mean age 53 | between day 2 and 5 post-myocardial infarction | Prospective case-control observational study. Portable diagnostic device (Somte; Compumedics, Australia) /No ESS | 64 | 94 | 68.1% |
| Loo 2014^34^ | Singapore/ sleep and stent study 8 countries | PCI | 670 M (85.4%)  115 F (14.6%) | Mean age 57.6 | prior to hospital discharge during the index PCI admission | Prospective case-control observational study. mbletta Gold standardized level-3 portable diagnostic device/BQ and ESS | 379 | 785 | 48.3% |
| Low 2013^35^ | Singapore/university affiliated hospital. | AMI | 143 M (89.4%) 17 F (10.6%) | Mean age 54.9 | Half in hospital and half post-discharge | Prospective case-control observational study. Portable diagnostic device (Embletta Gold, Natus Medical Inc., Canada) | 59 | 160 | 37% |
| Ludka 2014^19^ | Czech Republic/ 2 hospitals | AMI | 446 M (73.5%)  161 F (26.5%) | Mean age 62.0 | median duration from hospital admission to overnight sleep study was 4 days (range 2 to 14 days) | Prospective case-control observational study. Portable diagnostic device [Apnealink™ (ResMed) | 201 | 607 | 12.7% |
| Morra 2017^22^ | France/Coronary care unit | ACS | 74 M (73.5%) 27 F (26.5%) | Mean age 58.7 | Within 1-3 days of admission to ICU | Prospective case-control observational study. Portable ApneaLink ResMed/ESS | 46 | 101 | 45.5% |
| Planes 2010^36^ | France/ patient’s home` | ACS | 44 M (97.8%)  1 F (2.2%) | Mean age 63.4 | After PCI | Prospective case-control observational study. Type 3 portable CID102L8 /polysomnography (validation)/ESS | 31 | 45 | 69% |
| Shah 2013^37^ | USA/ Hospital | MI | 47 (60.3%) M 31 (39.7%) F | Mean age 58 | In hospital lstudied shortly after the onset of acute MI | Prospective case-control observational study. Apnealink Plus monitor type III portable device/ESS/BQ | 47 | 78 | 60.3% |
| Zeng 2019^38^ | China/ Hospital | ACS | 621 M  (82.6%) 131 (17.4%) F | Mean age 57 | After hospital admission | Prospective, single-center, observational cohort study. Portable Level 3 diagnostic device (Apnealink Air, Resmed, Australia) | 373 | 752 | 49.6% |
| Zhao 2015^39^ | Sigapore/university-affiliated hospital | ACS | 119 M (86.2%) 19 F (13.8%) | Mean age 61.8 | pre-operative evaluation of patients scheduled to undergo elective CABG | Watch-PAT 200 fourchannel unattended sleep monitoring device/BQ/ESS | 69 | 138 | 50% |
| Zhu 2017^40^ | China/Hospital | AMI | 80 (93%) M  6 (7%) F | Mean age 52 | 2 to 7 days after PCI | Prospective observational study. Portable diagnostic device (LS-100) | 40 | 86 | 46.5% |
| AHI 30 | | | | | | | | | |
| Hein 2013^41^ | Singapore/ 125 patients at tertiary institution | AMI | 122 M (97.6%) 3 F (2.4%) | Mean age 53.2 | Median 2 days after admission | Prospective case-control observational study. Level III portable diagnostic (Embletta Gold, ResMED)/ No ESS | 46 | 125 | 36.8% |
| Kiyokuni 2018^42^ | Japan/Hospital | ACS | 122 (79%) M  32 (21%) F | Mean age 66 | Within 7 days after admission | Apnomonitor Type 4 (SAS-2100, NIHON KOHDEN, Tokyo, Japan) | 33 | 154 | 21.4% |
| Ludka 2014^19^ | Czech Republic/ 2 hospitals | AMI | 446 M (73.5%)  161 F (26.5%) | Mean age 62.0 | median duration from hospital admission to overnight sleep study was 4 days (range 2 to 14 days) | Prospective case-control observational study. Portable diagnostic device [Apnealink™ (ResMed) | 77 | 607 | 12.7% |
| Morra 2017^22^ | France/Coronary care unit | ACS | 74 M 27 F | Mean age 58.7 | Within 1-3 days of admission to ICU | Prospective case-control observational study. Portable ApneaLink ResMed/ESS | 16 | 101 | 15.8% |
| Planes 2010^36^ | France/ patient’s home` | ACS | 44 M  1 F | Mean age 63.4 | After PCI | Prospective case-control observational study. Type 3 portable CID102L8 /polysomnography (validation)/ESS | 12 | 45 | 26.7% |
| Shah 2013^23^ | USA/ Hospital | MI | 47 M (60.3%) 31 F (39.7%) | Mean age 58 | In hospital studied shortly after the onset of acute MI | Apnealink Plus monitor type III portable device/ESS/BQ | 14 | 78 | 21.8% |

**References**

1. Garcia-Rio F, Alonso-Fernandez A, Armada E, et al. CPAP effect on recurrent episodes in patients with sleep apnea and myocardial infarction. International Journal of Cardiology 2013;168:1328-35.

2. Konecny T, Sert Kuniyoshi FH, Orban M, et al. Under-diagnosis of sleep apnea in patients after acute myocardial infarction. New York, New York: Elsevier Science; 2010:742-3.

3. Kuniyoshi FH, Garcia-Touchard A, Gami AS, et al. Day-night variation of acute myocardial infarction in obstructive sleep apnea. Journal of the American College of Cardiology 2008;52:343-6.

4. Leão S, Conde B, Fontes P, Calvo T, Afonso A, Moreira I. Effect of obstructive sleep apnea in acute coronary syndrome. American Journal of Cardiology 2016;117:1084-7.

5. Liu B, Guo R, Zhou S, Xie S, Wang K, Xu Y. Effects of obstructive sleep apnea on cardiac function and clinical outcomes in Chinese patients with ST-elevation myocardial infarction. Thescientificworldjournal 2014;2014:908582.

6. Nakashima H, Henmi T, Minami K, et al. Obstructive sleep apnoea increases the incidence of morning peak of onset in acute myocardial infarction. European Heart Journal Acute Cardiovascular Care 2013;2:153-8.

7. Sert Kuniyoshi FH, Zellmer MR, Calvin AD, et al. Diagnostic accuracy of the Berlin Questionnaire in detecting sleep-disordered breathing in patients with a recent myocardial infarction. Chest 2011;140:1192-7.

8. Buchner S, Eglseer M, Debl K, et al. Sleep disordered breathing and enlargement of the right heart after myocardial infarction. European Respiratory Journal 2015;45:680-90.

9. Danzi-Soares NJ, Genta PR, Nerbass FB, et al. Obstructive sleep apnea is common among patients referred for coronary artery bypass grafting and can be diagnosed by portable monitoring. Coronary artery disease 2012;23:31-8.

10. Furudono S, Kaibara S, Kurobe M, et al. Effects of sleep-disordered breathing on plaque characteristics in patients with acute myocardial infarction. European Heart Journal: Acute Cardiovascular Care 2015;4:169-70.

11. Kawashima C, Kiyokuni M, Narikawa M, et al. Severe sleep disordered breathing lead to renal dysfunction in patients with acute coronary syndrome underwent percutaneous coronary intervention. Circulation 2012;126.

12. Nakashima H, Katayama T, Takagi C, et al. Obstructive sleep apnoea inhibits the recovery of left ventricular function in patients with acute myocardial infarction. European Heart Journal 2006;27:2317-22.

13. Nakashima H, Kurobe M, Minami K, et al. Effects of moderate-to-severe obstructive sleep apnea on the clinical manifestations of plaque vulnerability and the progression of coronary atherosclerosis in patients with acute coronary syndrome. European Heart Journal: Acute Cardiovascular Care 2015;4:75-84.

14. Nakashima H, Muto S, Amenomori K, Shiraishi Y, Nunohiro T, Suzuki S. Impact of obstructive sleep apnea on myocardial tissue perfusion in patients with ST-segment elevation myocardial infarction. Circulation Journal 2011;75:890-6.

15. Aronson D, Nakhleh M, Zeidan-Shwiri T, Mutlak M, Lavie P, Lavie L. Clinical implications of sleep disordered breathing in acute myocardial infarction. PLoS ONE [Electronic Resource] 2014;9:e88878.

16. Berger S, Aronson D, Lavie P, Lavie L. Endothelial progenitor cells in acute myocardial infarction and sleep-disordered breathing. American Journal of Respiratory & Critical Care Medicine 2013;187:90-8.

17. Faria R, Areias V, Romero J, et al. Acute coronary syndrome and sleep apnea syndrome, a surprising association? European Heart Journal 2011;32:99.

18. Ishibashi Y, Osada N, Sekiduka H, et al. Peak time of acute coronary syndrome in patients with sleep disordered breathing. Journal of Cardiology 2009;53:164-70.

19. Ludka O, Stepanova R, Vyskocilova M, et al. Sleep apnea prevalence in acute myocardial infarction--the Sleep Apnea in Post-acute Myocardial Infarction Patients (SAPAMI) Study. International Journal of Cardiology 2014;176:13-9.

20. Mazaki T, Kasai T, Yokoi H, et al. Impact of sleep-disordered breathing on long-term outcomes in patients with acute coronary syndrome who have undergone primary percutaneous coronary intervention. Journal of the American Heart Association 2016;5.

21. Meng S, Fang L, Wang CQ, Wang LS, Chen MT, Huang XH. Impact of obstructive sleep apnoea on clinical characteristics and outcomes in patients with acute coronary syndrome following percutaneous coronary intervention. Journal of International Medical Research 2009;37:1343-53.

22. Morra S, Bughin F, Solecki K, et al. Prevalence of obstructive sleep apnoea in acute coronary syndrome: Routine screening in intensive coronary care units. Ann Cardiol Angeiol (Paris) 2017.

23. Shah N, Redline S, Yaggi HK, et al. Obstructive sleep apnea and acute myocardial infarction severity: ischemic preconditioning?.[Erratum appears in Sleep Breath. 2013 Sep;17(3):1119]. Sleep & Breathing 2013;17:819-26.

24. Ben Ahmed H, Boussaid H, Longo S, et al. Impact of obstructive sleep apnea in recruitment of coronary collaterality during inaugural acute myocardial infarction. Annales de Cardiologie et d Angeiologie 2015;64:273-8.

25. de Batlle J, Turino C, Sanchez-de-la-Torre A, et al. Predictors of obstructive sleep apnoea in patients admitted for acute coronary syndrome. Eur Respir J 2017;49.

26. Fan J, Wang X, Ma X, Somers VK, Nie S, Wei Y. Association of Obstructive Sleep Apnea With Cardiovascular Outcomes in Patients With Acute Coronary Syndrome. J Am Heart Assoc 2019;8:e010826.

27. Florés M, Sánchez-de-la-Torre M, Esquinas C, et al. Effect of obstructive sleep apnea in severity and short-term prognosis of acute coronary syndrome. European Respiratory Journal 2014;44.

28. Fox H, Purucker HC, Holzhacker I, et al. Prevalence of sleep-disordered breathing and patient characteristics in a coronary artery disease cohort undergoing cardiovascular rehabilitation. Journal of Cardiopulmonary Rehabilitation and Prevention 2016;36:421-9.

29. Hayashi H, Fukuma N, Kato K, Kato Y, Takahashi H, Mizuno K. Clinical backgrounds and the time course of sleep-disordered breathing in patients after myocardial infarction. Journal of Nippon Medical School = Nihon Ika Daigaku Zasshi 2013;80:192-9.

30. Jiang XM, Qian XS, Gao XF, et al. Obstructive Sleep Apnea Affecting Platelet Reactivity in Patients Undergoing Percutaneous Coronary Intervention. Chin Med J (Engl) 2018;131:1023-9.

31. Koo CY, de la Torre AS, Loo G, et al. Effects of Ethnicity on the Prevalence of Obstructive Sleep Apnoea in Patients with Acute Coronary Syndrome: A Pooled Analysis of the ISAACC Trial and Sleep and Stent Study. Heart Lung and Circulation 2016.

32. Lee CH, Khoo SM, Tai BC, et al. Obstructive sleep apnea in patients admitted for acute myocardial infarction. Prevalence, predictors, and effect on microvascular perfusion. Chest 2009;135:1488-95.

33. Lee LC, Torres MC, Khoo SM, et al. The relative impact of obstructive sleep apnea and hypertension on the structural and functional changes of the thoracic aorta. Sleep 2010;33:1173-6.

34. Loo G, Koo CY, Zhang J, et al. Impact of obstructive sleep apnea on cardiovascular outcomes in patients treated with percutaneous coronary intervention: rationale and design of the sleep and stent study. Clinical Cardiology 2014;37:261-9.

35. Low TT, Hong WZ, Tai BC, et al. The influence of timing of polysomnography on diagnosis of obstructive sleep apnea in patients presenting with acute myocardial infarction and stable coronary artery disease. Sleep Medicine 2013;14:985-90.

36. Planes C, Leroy M, Bouach Khalil N, et al. Home diagnosis of obstructive sleep apnoea in coronary patients: validity of a simplified device automated analysis. Sleep & Breathing 2010;14:25-32.

37. Shah N, Redline S, Yaggi HK, et al. Obstructive sleep apnea and acute myocardial infarction severity: ischemic preconditioning? Sleep Breath 2013;17:819-26.

38. Zeng Y, Yang S, Wang X, Fan J, Nie S, Wei Y. Prognostic impact of residual SYNTAX score in patients with obstructive sleep apnea and acute coronary syndrome: a prospective cohort study. Respir Res 2019;20:43.

39. Zhao LP, Kofidis T, Chan SP, et al. Sleep apnoea and unscheduled re-admission in patients undergoing coronary artery bypass surgery. Atherosclerosis 2015;242:128-34.

40. Zhu CP, Li TP, Wang X, et al. The relationship between apnoea hypopnoea index and Gensini score in patients with acute myocardial infarction undergoing emergency primary percutaneous coronary intervention. J Thorac Dis 2017;9:2476-83.

41. Hein T, Loo G, Ng WY, et al. Relationship between apnoea-hypopnoea index and angiographiccoronary disease phenotypes in patients presenting with acutemyocardial infarction. Acute Cardiac Care 2013;15:26-33.

42. Kiyokuni M, Kawashima C, Konishi M, et al. Relationship between sleep-disordered breathing and renal dysfunction in acute coronary syndrome. J Cardiol 2018;71:168-73.
